# Delay in Celiac Disease Diagnosis Among Patients with High-Risk Screening Conditions: Results from a United States Claims Database

**DOI:** 10.3390/jcm14186471

**Published:** 2025-09-14

**Authors:** Haley M. Zylberberg, Erin B. P. Miller, Deirdre Reidy, Kate Avery, Carolyn Newberry, Amy Ratner, Debra G. Silberg

**Affiliations:** 1 Division of Gastroenterology and Hepatology, Weill Cornell Medicine, New York, NY 10065, USA; hmz7001@med.cornell.edu (H.M.Z.); can9054@med.cornell.edu (C.N.); 2 Beyond Celiac, Ambler, PA 19002, USA; emiller@beyondceliac.org (E.B.P.M.); kavery@beyondceliac.org (K.A.); aratner@beyondceliac.org (A.R.); 3 Department of Medicine, Weill Cornell Medicine, New York, NY 10065, USA; dif9035@nyp.org

**Keywords:** celiac disease, diagnosis

## Abstract

**Introduction:** Celiac disease (CeD) incidence has risen in the past two decades, but delays in diagnosis continue. The aim of this study is to evaluate sociodemographic factors associated with time to diagnosis in individuals at high risk for CeD in the United States (US). **Methods:** We performed a retrospective analysis using a random 25% sample of US private payer health insurance claims from 2007 to 2022. Patients with preceding high-risk conditions for CeD, as defined by professional societies, were identified. From this cohort, patients with CeD (ages >1 year) were selected. Time to diagnosis was calculated from the date of the high-risk condition to the date of biopsy via endoscopy. Associations between time to diagnosis and covariates were evaluated using descriptive statistics, including mean with standard deviation (SD), *t* tests, ANOVA, and Tukey’s HSD tests where applicable. **Results:** We identified 9344 patients with CeD with a high-risk condition preceding diagnosis. The mean time to diagnosis was about 3 years (SD 36.8), with a median of 20.4 months (range < 1 month to 190.2 months). The mean time to diagnosis was longer in females by 3.7 months compared to males (*p* < 0.0001). The average time to diagnosis for older adults aged 61–100 years was 9 months longer than that for adults aged 21–60 years and 18 months longer than that for children (*p* < 0.0001). Differences in mean time to diagnosis also varied by race and region (*p* < 0.0001). **Conclusions**: Despite the presence of high-risk conditions that typically call for heightened screening, CeD diagnosis is frequently delayed for years. These findings highlight that enhancing awareness of CeD remains essential for promoting timely diagnosis, especially in vulnerable populations.

## 1. Introduction

Celiac disease (CeD) is a chronic autoimmune disease caused by gluten ingestion in individuals with genetic susceptibility [1,2]. Despite increasing incidence, awareness, and improvements in diagnostic tools, significant delays in CeD diagnosis persist [1,2,3,4]. Research indicates that there can be a wait of several months to years between the onset of symptoms and an eventual diagnosis, with some studies finding a delay of more than 10 years in some individuals [5,6,7,8,9,10,11,12,13,14,15,16]. Of these studies, few have been performed in the United States (US) or within the past decade [7,12,16].

Diagnosis of CeD begins with serologic testing, most commonly anti-tissue transglutaminase Immunoglobulin A (anti-TTG IgA), which has high sensitivity and specificity [17]. Confirmation with duodenal biopsy on upper endoscopy is considered the gold standard [17]. In Europe, particularly in children, guidelines permit a non-biopsy pathway when anti-TTG IgA levels are ≥10× the upper limit of normal and confirmed with endomysial antibody on a separate occasion [18]. In contrast, the North American Society for Pediatric Gastroenterology, Hepatology and Nutrition (NASPGHAN) recommends biopsy because of assay variability and risk of false positive results [19]. In the most recent guidelines, the American College of Gastroenterology (ACG) favors duodenal biopsy, but allows for a non-biopsy approach in select children [17].

Currently, there is no universal recommendation for CeD screening in the US [20]. However, medical society guidelines outline high-risk symptoms and conditions that should prompt providers to screen for CeD [17,19,21,22,23,24]. These include gastrointestinal symptoms such as chronic diarrhea and recurrent abdominal pain, extra-intestinal symptoms such as failure to thrive and anemia, as well as a family history of CeD, and related conditions such as type 1 diabetes, among others [17,19,21,22,23]. While children are more likely to present with the classical manifestations, including diarrhea, weight loss, and malabsorption, the majority of adults present with extra-intestinal symptoms [25]. Still, fatigue, short stature, and headache are common symptoms of CeD in children [26]. These atypical symptoms may be less well-recognized by providers and lead to a prolonged time to diagnosis. Studies point to both patient and provider explanations for delays in diagnosis [8,9,11,15,16]. Long waits for diagnosis of CeD cause prolongation of untreated symptoms and manifestations, increased severity of disease, poor quality of life, and increased healthcare costs [4,10,11,15,27,28]. Therefore, the aim of this study is to evaluate the time to diagnosis of CeD in a large nationwide US population and to assess sociodemographic factors associated with time to diagnosis.

## 2. Methods

We conducted a retrospective analysis of CeD patients spanning 2007–2022 from the Optum Clinformatics^®^ Data Mart, a large nationally representative US private payer health insurance claims database [29]. Data were provided to our study team from the Data Mart in a de-identified format at the final stage of cohort selection.

The initial study population consisted of a random 25% sample from the Data Mart, which was utilized to facilitate analysis given the large dataset size. From this sample, patients were identified with at least 1 claim (defined by International Classification of Diseases (ICD) 9 or 10 code) for a high-risk clinical symptom or medical condition recommended for CeD screening by clinical guidelines [17,19,21,22,23]. These high-risk conditions included the following: recurrent abdominal pain; ataxia; autoimmune thyroid disease; chronic constipation, unexplained; dental enamel defects; dermatitis herpetiformis; diarrhea, chronic; Down syndrome; failure to thrive; hypertransaminasemia, cryptogenic; intestinal malabsorption, unspecified; iron deficiency anemia; irritable bowel syndrome; oral aphthous ulcers, severe or persistent; osteomalacia or premature osteoporosis; peripheral neuropathy; short stature; Turner syndrome; type 1 diabetes; weight loss, unexplained; Williams syndrome (Supplemental Table S1). The index date was defined as the date of the first occurrence of the ICD code for the high-risk condition. Among those with a high-risk screening condition, our final cohort of patients included those >1 year old with an ICD code for CeD (defined by 579.0 or K90.0) after the index date. These patients additionally needed to have undergone CeD screening (defined by the presence of serologic testing, including tissue transglutaminase antibody, endomysial antibody, gliadin deamidated antibody, or biopsy for dermatitis herpetiformis) and upper endoscopy with biopsy after the index date and before the ICD code for CeD. Exclusion criteria included all patients with a diagnosis of CeD prior to the index date or who had CeD screening tests or upper endoscopy with biopsy before or on the same date as the index date. Inclusion and exclusion criteria were applied after the random 25% sample was generated.

Demographic variables included region, race/ethnicity, gender, and age, which were directly pulled from the Data Mart. Region, gender, and race/ethnicity were determined at the time of insurance enrollment, while age was determined at the time of index date. Age groups were divided into children (1 to 20 years), adults (21 to 60), and older adults (61 to 100), as well as by 10-year age groups. Regions were divided into Midwest, Northeast, South, West, and unknown.

The primary outcome of interest was time to diagnosis of CeD, which was calculated as the time from the index date of the high-risk condition to the confirmed diagnosis by date of upper endoscopy with biopsy. Secondary outcomes included differences in time to diagnosis by age group, gender, race, geographic region, and high-risk conditions.

Sociodemographic and clinical variables were summarized using counts with percentages, mean with standard deviation (SD), and median with interquartile range (IQR). Differences between time to diagnosis for each variable were assessed using *t* tests, ANOVA, and Tukey’s HSD tests where applicable, with a two-sided *p* value of <0.05 considered significant. The mean time to diagnosis was compared, given the de-identified nature of the data. Each high-risk screening condition was compared against the presence of at least one other high-risk screening condition, as all patients in the cohort had at least one condition. This study was deemed to be exempt from IRB review by North Star IRB, given the use of de-identified data (Protocol #NB400235).

## 3. Results

Overall, 9344 patients with CeD who had a preceding high-risk condition were identified (Table 1 and Table 2). The most common high-risk conditions were recurrent abdominal pain (75.1%) and chronic diarrhea (62.4%). Most patients were female (72.5%), White (79.7%), aged 21–60 years (55.9%), and from the Southern US (35.7%).

**Figure 1:**
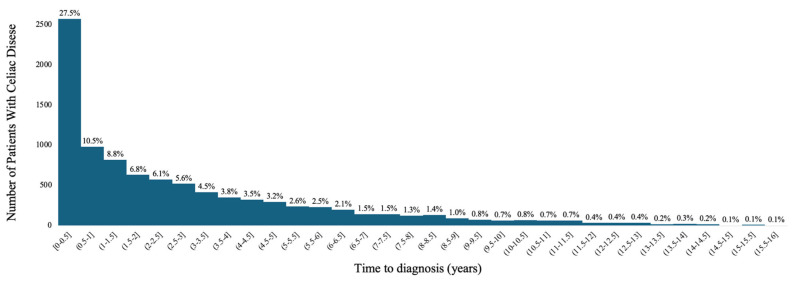
Time to Diagnosis Among Patients with Celiac Disease.

The mean time to diagnosis was approximately 3 years (33.7 months, SD 36.8). Figure 1 shows the wide range in time to diagnosis from <1 month to almost 16 years (190.2 months). Slightly more than one-third of patients were diagnosed within the first year of presenting with a high-risk condition; 27.5% were diagnosed within the first 6 months, and an additional 10.5% were diagnosed between 6 months and 1 year. Fifty percent of patients were diagnosed within 2 years of a documented high-risk condition (median 20.4 months, IQR 4.9–50.6 months), and 90% of patients were diagnosed within 7 years (88.8 months).

Table 1 shows the time to diagnosis by sociodemographic variables. Female patients had a longer mean time to diagnosis compared to male patients by 3.7 months (*p* < 0.0001). The median time to diagnosis was 21.8 months in women (IQR 5.5–52.5) compared to 16.8 months (IQR 3.94–5.4) in men. Older adults aged 61–100 years on average took 9 months longer to be diagnosed with CeD (41.7 months, SD 38.4) compared to adults aged 21–60 years (32.5 months, SD 36.7) and 18 months longer compared to children (24.1 months, SD 31.4, *p* < 0.0001). When evaluating patients by 10-year age groups, older adults aged 81–90 years had the longest mean time to diagnosis (51.5 months, SD 40.8) compared to all younger age groups (*p* < 0.0001). Time to diagnosis was an average of 2.8 years longer for older adults aged 81–90 than for children aged 1–10 (33.7 months, 95% CI [25.7–41.7 months], *p* < 0.0001). Mean time to diagnosis differed significantly by race (*p* < 0.0001). This difference was attributable to Hispanic patients, who experienced an average diagnostic delay of four months longer than non-Hispanic White patients (*p* = 0.0452). Both the overall (*p* = 0.0311) and specific (*p* = 0.0330) comparisons remained significant when unknown race was excluded.

Mean time to diagnosis also differed significantly by US region (*p* < 0.0001). Patients in the South had a mean time to diagnosis 4.7 months longer than those in the Midwest and 5.8 months longer than those in the Northeast (*p* < 0.0001), though no statistically significant difference was seen compared to patients in the West. In addition to reduced time to diagnosis compared to the South, the Northeast also had, on average, a shorter diagnosis time by 3.9 months compared to the West (*p* = 0.0103), with no difference seen when compared to the Midwest.

All high-risk conditions showed a mean time to diagnosis of >1.7 years. Table 2 shows the mean time to diagnosis for each condition compared to the presence of at least one other condition, given that all patients in the cohort had a high-risk screening condition. At 20.7 months, only failure to thrive had a significantly shorter mean time to diagnosis compared to its absence (and presence of any other condition) (*p* < 0.0001).

## 4. Discussion

In this nationwide US study of 9344 people with CeD, the average time to diagnosis was almost three years. Despite recommendations from major medical societies to screen patients with high-risk symptoms and conditions [17,19,21,22,23], our study found that long times to diagnosis of CeD persist in certain groups and highlights a gap in adherence to these guidelines. These findings support consideration of universal screening of CeD or increased clinician attention and awareness to improve screening adherence in high-risk populations.

While most previous studies on this topic have been single-center or regional cohorts, this analysis is a large nationwide US study using claims data. Only one previous study included a nationwide US population, but it was conducted more than 20 years ago and is based on survey data, which can be limited by recall bias and lack of clinical verification [16]. That study found a time to diagnosis of 12 months [16]. A 2017 Illinois-based study found a time to diagnosis of 2.3 months for gastrointestinal symptoms and 42 months for non-gastrointestinal symptoms [12]. Meanwhile, a 2016 New York-based study found a mean time to diagnosis of 11 years, with the majority of patients having diarrhea [7]. Time to diagnosis reported in non-US studies also varies widely, with the shortest time to diagnosis observed in Italy (on the order of months) [9], and the longest in studies from Sweden, Finland, Denmark, and Canada (upwards of 10 years) [6,11,13]. Differences in healthcare systems between Europe and the US may also influence diagnostic timelines, as access in the US is mediated by insurance coverage, whereas in most European countries, healthcare is government-provided and broadly accessible at low cost. Nevertheless, studies outside of the US still report prolonged times to celiac diagnosis [6,11,13].

Compared to adults, children had a shorter time to CeD diagnosis. This is in keeping with a recent Italian study showing short times to diagnosis in children, though this study found a mean time to diagnosis of only 5 months [30]. In our study, children’s relatively shorter time to diagnosis may be partly explained by the presence of failure to thrive, which was also associated with a reduced time to diagnosis. Given its clinical urgency in pediatric patients, failure to thrive may prompt timely investigation compared to other symptoms. This is in contrast, however, to the Italian study showing a longer time to diagnosis in children with failure to thrive [30], which may be explained by different practice patterns in each country. Unfortunately, we could not quantify the number of children with failure to thrive in this cohort.

In contrast to children and younger adults, older adults had the longest time to diagnosis. This may be due to the absence of gastrointestinal symptoms seen in this population [31], which has been found to be associated with longer times to diagnosis in some studies [12,27]. Further, non-gastrointestinal symptoms in older adults, such as anemia or osteoporosis, may be misattributed solely to aging or to comorbidities of other chronic conditions [31,32]. Older adults may also be less likely to undergo upper endoscopy due to comorbidities or contraindications to the procedure, which impact diagnosis. Additionally, even when upper endoscopy is performed, older adults are also less likely to undergo duodenal biopsies compared to younger adults, which suggests that gastroenterologists are not considering CeD in this population [33]. Since all patients in our study underwent upper endoscopy with biopsy, the lack of duodenal sampling would not explain the longer time to diagnosis we found in older adults, and the actual average time to diagnosis may be longer. The increasing incidence of CeD in older adults [3,34], coupled with the long time to diagnosis in our study, highlights that special attention to the older patient population with CeD is imperative. Unfortunately, we were unable to evaluate specific symptoms by age and therefore could not assess whether time to diagnosis differed by symptom.

Although CeD is more commonly diagnosed in women than men [2], women experience a longer time to diagnosis. While this study found a significant average difference of 3.7 months, other research in European cohorts has found upwards of 30-month differences in time to diagnosis between genders [6,15]. Women may be more likely to have their symptoms attributed to functional disorders, such as irritable bowel syndrome (IBS), rather than CeD [5,6,15]. Previous research in Switzerland has examined this gender difference, and data suggest that IBS alone cannot explain the long time to diagnosis; rather, a driving force is due to clinician delays in appropriate testing [15]. Unfortunately, we were unable to evaluate changes in diagnosis over time, such as those potentially influenced by the introduction of CeD screening guidelines, which may impact provider testing [17,19,21,22,23]. Nevertheless, long waits for diagnosis are not unique to CeD; women have also been shown to experience delays or underdiagnosis in other gastrointestinal disorders, such as inflammatory bowel disease [35]. Delays in diagnosis across diseases may be partly driven by the tendency to dismiss or downplay women’s health complaints, specifically pain [36]. Further research is needed to understand the factors contributing to longer time to diagnosis in women with CeD.

In regard to racial/ethnic disparities, our analysis found that Hispanic patients experienced a significantly longer time to diagnosis by 4 months compared to non-Hispanic White patients, while other racial groups had comparable times to diagnosis. This could be due to misconceptions and under-recognition of CeD in non-White populations. For example, lower rates of duodenal biopsies are performed in the US Hispanic and Black populations [33]. Additionally, a previous US study of Medicare data performed by our group found that CeD relative prevalence inversely correlated with Latino/Hispanic ethnicity, Black race, and increasing social deprivation [37]. Additional barriers may arise from communication challenges, as many Hispanic Americans report difficulties related to language barriers and feeling the need to advocate for themselves to receive appropriate care [38]. Our findings thus raise questions about systemic or access-related barriers that may disproportionately affect Hispanic individuals with CeD.

Regional differences were also significant in this analysis, with patients in the Northeast diagnosed more quickly than those in the South and West. This difference may be attributed to the distribution of celiac-specific specialty centers, with four recognized centers in the Northeast and more limited access in other areas of the country [39]. Our previous study found that CeD relative prevalence was positively correlated with proximity to a celiac center, suggesting that geographic expertise may play a role in clinician awareness and timeliness of diagnosis [37].

Strengths of our study include the use of medical data from a large, nationwide US sample encompassing a diversity of ages and races over a 15-year period, providing a comprehensive assessment of diagnostic patterns across the country. This study has several limitations, primarily related to the use of private insurance claims data, which does not include individuals who were uninsured or covered by public programs. ICD-9 and ICD-10 codes may be subject to misclassification, especially for nonspecific conditions such as abdominal pain, where repeated coding may not accurately reflect chronic or recurrent symptoms. Additionally, the dataset was provided to us in a de-identified format, which precluded us from performing more detailed subgroup and temporal analyses, such as evaluation of time to diagnosis before and after different screening guideline publications or further delineation by age, sex, and symptoms. Our dataset also lacked insurance and socioeconomic data, which may influence time to diagnosis and warrant further study. Lastly, it is important to note that the overall differences in time to diagnosis between certain groups were relatively small, generally on the order of a few months, which may limit the clinical significance of some of these findings. Because only means and standard deviations were available, nonparametric testing could not be performed, and results should be interpreted with this limitation in mind.

Overall, our analysis reveals persistent delays in CeD diagnosis across the US, supporting the need for renewed efforts to close diagnostic gaps, especially among underserved and vulnerable populations. Targeted strategies, including provider education, increased access to specialty care, and consideration of universal screening, may help address these ongoing disparities.

## Figures and Tables

**Table 1:** Mean Time to Celiac Disease Diagnosis by Sociodemographic Variables.

Variable	*N* (%)	Mean Time in Months (Standard Deviation)	*p* Value
Gender:			<0.0001
Female	6771 (72.46)	34.74 (37.08)	
Male	2571 (27.51)	31.08 (36.08)	
Age:			<0.0001
1–20 years	1488 (15.92)	24.11 (31.39)	
21–60 years	5225 (55.92)	32.49 (36.70)	
61–100 years	2630 (28.15)	41.65 (38.35)	
Region:			<0.0001
Midwest	2138 (22.88)	31.65 (36.10)	
Northeast	1832 (19.61)	30.59 (33.21)	
South	3337 (35.71)	36.35 (38.81)	
West	2013 (21.54)	34.44 (36.94)	
Unknown	24 (0.26)	37.32 (47.75)	
Race:			<0.0001
Asian, non-Hispanic	185 (1.98)	32.41 (35.48)	
Black, non-Hispanic	430 (4.60)	36.30 (38.22)	
Hispanic	685 (7.33)	38.45 (37.79)	
White, non-Hispanic	7447 (79.70)	34.41 (37.13)	
Unknown	597 (6.39)	18.52 (26.56)	

**Table 2:** Mean Time (in months) to Celiac Disease Diagnosis by High-Risk Screening Conditions.

High Risk Screening Condition	Presence		Absence		*p* Value
	N (%)	Mean (SD)	N (%)	Mean (SD)	
Abdominal pain (recurrent)	7018 (75.11)	36.20 (37.89)	2326 (24.89)	26.30 (32.36)	<0.0001
Ataxia	1007 (10.78)	49.13 (40.58)	8337 (89.22)	31.88 (35.92)	<0.0001
Autoimmune thyroid disease	868 (9.29)	44.43 (39.43)	8476 (90.71)	32.65 (36.39)	<0.0001
Constipation (chronic, unexplained)	376 (4.02)	42.09 (39.11)	8968 (95.98)	33.39 (36.70)	<0.0001
Dental enamel defects	0 (0.00)	-	9344 (100.00)	-	-
Dermatitis herpetiformis	238 (2.55)	30.78 (33.17)	9106 (97.45)	33.82 (36.93)	0.2089
Diarrhea (chronic)	5833 (62.43)	35.72 (37.32)	3511 (37.57)	30.46 (35.80)	<0.0001
Down syndrome	46 (0.49)	38.55 (41.03)	9298 (99.51)	33.72 (36.82)	0.3751
Failure to thrive	233 (2.49)	20.69 (28.68)	9111 (97.51)	34.07 (36.97)	<0.0001
Family history	471 (5.04)	30.96 (38.14)	8873 (94.96)	33.89 (36.77)	0.0926
Hypertransaminasemia (cryptogenic)	183 (1.96)	36.67 (39.59)	9161 (98.04)	33.68 (36.78)	0.2770
Intestinal malabsorption (unspecified)	859 (9.19)	35.59 (37.47)	8485 (90.81)	33.55 (36.77)	0.1220
Iron deficiency anemia	2568 (27.48)	39.75 (38.23)	6776 (72.52)	31.46 (36.04)	<0.0001
Irritable bowel syndrome	2662 (28.49)	40.42 (38.03)	6682 (71.51)	31.08 (36.02)	<0.0001
Oral aphthous ulcers (severe or persistent)	249 (3.84)	48.79 (41.90)	9095 (97.34)	33.33 (36.61)	<0.0001
Osteomalacia or premature osteoporosis	359 (1.56)	42.46 (37.38)	8985 (96.16)	33.39 (36.78)	<0.0001
Peripheral neuropathy	146 (2.75)	46.57 (41.18)	9198 (98.44)	33.54 (36.73)	<0.0001
Short stature	257 (0.21)	30.06 (37.86)	9087 (97.25)	33.84 (36.81)	0.1048
Turner syndrome	20 (6.88)	37.34 (33.02)	9324 (99.79)	33.73 (36.85)	0.6616
Type 1 diabetes	643 (6.88)	43.25 (37.22)	8701 (93.12)	33.04 (36.72)	<0.0001
Weight loss (unexplained)	2357 (25.22)	37.58 (38.32)	6987 (74.78)	32.45 (36.24)	<0.0001
Williams syndrome	0 (0.0)	-	9344 (100.00)	-	-

## Data Availability

The data underlying this study are not publicly available due to licensing restrictions from the data provider and cannot be shared by the authors.

## Supplementary Materials

The following supporting information can be downloaded at: https://www.mdpi.com/article/10.3390/jcm14186471/s1, Table S1: ICD Codes by Symptom or Condition.

## Abbreviations

CeD	Celiac Disease
IBS	Irritable Bowel Syndrome
IQR	Interquartile Range
TTG IgA	Tissue Transglutaminase Immunoglobulin A
SD	Standard Deviation
US	United States
