# Supplementary material for: Delay in Celiac Disease Diagnosis Among Patients with High-Risk Screening Conditions: Results from a United States Claims Database
Source: J Clin Med. 2025 Sep 14;14(18):6471. doi: 10.3390/jcm14186471 (PMC12470924; doi:10.3390/jcm14186471)
Supplement: Supplementary file 1 [file jcm-14-06471-s001.zip › jcm-3803879-supplementary.pdf]

Supplemental Table S1. ICD Codes by Symptom or Condition

**Abdominal pain, recurrent** ***(any 2+ codes in a 12-month period)***	
ICD-9 789.00	ICD-9 789.09
ICD-9 789.07	ICD-10 R10.9
ICD-10 R10.10	ICD-10 R10.30
ICD-10 R10.84	
**Ataxia**	
ICD-9 781.2	ICD-9 781.3
ICD-10 R26.0	ICD-10 R27.0
ICD-10 R27.8	ICD-10 R27.9
**Autoimmune thyroid disease**	
ICD-9 245.2	ICD-10 E06.3
**Celiac disease**	
ICD-9 579.0	ICD-10 K90.0
**Chronic constipation, unexplained**	
ICD-9 564.09	ICD-10 K59.04
**Dental enamel defects**	
ICD-9 520.4	ICD-9 520.8
ICD-10 K00.4	ICD-10 K00.8
**Dermatitis herpetiformis**	
ICD-9 694.0	ICD-10 L13.0
**Diarrhea, chronic**	
ICD-9 787.91	ICD-10 R19.7
**Down syndrome**	
ICD-9 758.0	ICD-10 Q90.0
ICD-10 Q90.1	ICD-10 Q90.2
ICD-10 Q90.9	
**Failure to thrive**	
ICD-9 783.41	ICD-10 R62.51
**First-degree relative with celiac disease**	
ICD-9 V18.59	ICD-10 Z83.79
**Hypertransaminasemia, cryptogenic**	
ICD-10 R74.01	
**Intestinal malabsorption, unspecified**	
ICD-9 579.9	ICD-10 K90.9
**Iron deficiency anemia**	
ICD-9 280.9	ICD-10 D50.9
**Irritable bowel syndrome**	
ICD-9 564.1	ICD-10 K58.0
ICD-10 K58.1	ICD-10 K58.2
ICD-10 K58.8	ICD-10 K58.9
**Oral aphthous ulcers, severe or persistent**	
ICD-9 528.2	ICD-10 K12.0
**Osteomalacia or premature osteoporosis**	
ICD-9 733.02	ICD-9 268.2

ICD-10 M81.8
ICD-10 M80.80XD
ICD-10 M80.80XK
ICD-10 M80.80XS
ICD-10 M80.811D
ICD-10 M80.811K
ICD-10 M80.811S
ICD-10 M80.812D
ICD-10 M80.812K
ICD-10 M80.812S
ICD-10 M80.819D
ICD-10 M80.819K
ICD-10 M80.819S
ICD-10 M80.821D
ICD-10 M80.821K
ICD-10 M80.821S
ICD-10 M80.822D
ICD-10 M80.822K
ICD-10 M80.822S
ICD-10 M80.829D
ICD-10 M80.829K
ICD-10 M80.829S
ICD-10 M80.831D
ICD-10 M80.831K
ICD-10 M80.831S
ICD-10 M80.832D
ICD-10 M80.832K
ICD-10 M80.832S
ICD-10 M80.839D
ICD-10 M80.839K
ICD-10 M80.839S
ICD-10 M80.841D
ICD-10 M80.841K
ICD-10 M80.841S
ICD-10 M80.842D
ICD-10 M80.842K
ICD-10 M80.842S
ICD-10 M80.849D
ICD-10 M80.849K
ICD-10 M80.849S
ICD-10 M80.851D
ICD-10 M80.851K
ICD-10 M80.851S
ICD-10 M80.852D
ICD-10 M80.852K
ICD-10 M80.852S

ICD-10 M80.80XA
ICD-10 M80.80XG
ICD-10 M80.80XP
ICD-10 M80.811A
ICD-10 M80.811G
ICD-10 M80.811P
ICD-10 M80.812A
ICD-10 M80.812G
ICD-10 M80.812P
ICD-10 M80.819A
ICD-10 M80.819G
ICD-10 M80.819P
ICD-10 M80.821A
ICD-10 M80.821G
ICD-10 M80.821P
ICD-10 M80.822A
ICD-10 M80.822G
ICD-10 M80.822P
ICD-10 M80.829A
ICD-10 M80.829G
ICD-10 M80.829P
ICD-10 M80.831A
ICD-10 M80.831G
ICD-10 M80.831P
ICD-10 M80.832A
ICD-10 M80.832G
ICD-10 M80.832P
ICD-10 M80.839A
ICD-10 M80.839G
ICD-10 M80.839P
ICD-10 M80.841A
ICD-10 M80.841G
ICD-10 M80.841P
ICD-10 M80.842A
ICD-10 M80.842G
ICD-10 M80.842P
ICD-10 M80.849A
ICD-10 M80.849G
ICD-10 M80.849P
ICD-10 M80.851A
ICD-10 M80.851G
ICD-10 M80.851P
ICD-10 M80.852A
ICD-10 M80.852G
ICD-10 M80.852P
ICD-10 M80.859A

ICD-10 M80.859D	ICD-10 M80.859G
ICD-10 M80.859K	ICD-10 M80.859P
ICD-10 M80.859S	ICD-10 M80.861A
ICD-10 M80.861D	ICD-10 M80.861G
ICD-10 M80.861K	ICD-10 M80.861P
ICD-10 M80.861S	ICD-10 M80.862A
ICD-10 M80.862D	ICD-10 M80.862G
ICD-10 M80.862K	ICD-10 M80.862P
ICD-10 M80.862S	ICD-10 M80.869A
ICD-10 M80.869D	ICD-10 M80.869G
ICD-10 M80.869K	ICD-10 M80.869P
ICD-10 M80.869S	ICD-10 M80.871A
ICD-10 M80.871D	ICD-10 M80.871G
ICD-10 M80.871K	ICD-10 M80.871P
ICD-10 M80.871S	ICD-10 M80.872A
ICD-10 M80.872D	ICD-10 M80.872G
ICD-10 M80.872K	ICD-10 M80.872P
ICD-10 M80.872S	ICD-10 M80.879A
ICD-10 M80.879D	ICD-10 M80.879G
ICD-10 M80.879K	ICD-10 M80.879P
ICD-10 M80.879S	ICD-10 M80.88XA
ICD-10 M80.88XD	ICD-10 M80.88XG
ICD-10 M80.88XK	ICD-10 M80.88XP
ICD-10 M80.88XS	ICD-10 M80.8AXA
ICD-10 M80.8AXD	ICD-10 M80.8AXG
ICD-10 M80.8AXK	ICD-10 M80.8AXP
ICD-10 M80.8AXS	ICD-10 M83.9
**Peripheral neuropathy**	
ICD-9 337.00	ICD-10 G90.09
**Short stature**	
ICD-9 783.43	ICD-10 R62.52
**Turner syndrome**	
ICD-9 758.6	ICD-10 Q96.0
ICD-10 Q96.1	ICD-10 Q96.2
ICD-10 Q96.3	ICD-10 Q96.4
ICD-10 Q96.8	ICD-10 Q96.9
**Type 1 diabetes**	
ICD-9 250.11	ICD-9 250.31
ICD-9 250.41	ICD-9 250.51
ICD-9 250.61	ICD-9 250.71
ICD-9 250.81	ICD-9 250.31
ICD-9 250.91	ICD-9 250.01
ICD-10 E10.10	ICD-10 E10.11
ICD-10 E10.21	ICD-10 E10.22
ICD-10 E10.29	ICD-10 E10.311
ICD-10 E10.319	ICD-10 E10.3211

ICD-10 E10.3212	ICD-10 E10.3213
ICD-10 E10.3219	ICD-10 E10.3291
ICD-10 E10.3292	ICD-10 E10.3293
ICD-10 E10.3299	ICD-10 E10.3311
ICD-10 E10.3312	ICD-10 E10.3313
ICD-10 E10.3319	ICD-10 E10.3391
ICD-10 E10.3392	ICD-10 E10.3393
ICD-10 E10.3399	ICD-10 E10.3411
ICD-10 E10.3412	ICD-10 E10.3413
ICD-10 E10.3419	ICD-10 E10.3491
ICD-10 E10.3492	ICD-10 E10.3493
ICD-10 E10.3499	ICD-10 E10.3511
ICD-10 E10.3512	ICD-10 E10.3513
ICD-10 E10.3519	ICD-10 E10.3521
ICD-10 E10.3522	ICD-10 E10.3523
ICD-10 E10.3529	ICD-10 E10.3531
ICD-10 E10.3532	ICD-10 E10.3533
ICD-10 E10.3539	ICD-10 E10.3541
ICD-10 E10.3542	ICD-10 E10.3543
ICD-10 E10.3549	ICD-10 E10.3551
ICD-10 E10.3552	ICD-10 E10.3553
ICD-10 E10.3559	ICD-10 E10.3591
ICD-10 E10.3592	ICD-10 E10.3593
ICD-10 E10.3599	ICD-10 E10.36
ICD-10 E10.37X1	ICD-10 E10.37X2
ICD-10 E10.37X3	ICD-10 E10.37X9
ICD-10 E10.39	ICD-10 E10.40
ICD-10 E10.41	ICD-10 E10.42
ICD-10 E10.43	ICD-10 E10.44
ICD-10 E10.49	ICD-10 E10.51
ICD-10 E10.52	ICD-10 E10.59
ICD-10 E10.610	ICD-10 E10.618
ICD-10 E10.620	ICD-10 E10.621
ICD-10 E10.622	ICD-10 E10.628
ICD-10 E10.630	ICD-10 E10.638
ICD-10 E10.641	ICD-10 E10.649
ICD-10 E10.65	ICD-10 E10.69
ICD-10 E10.8	ICD-10 E10.9
**Weight loss, unexplained**	
ICD-9 783.21	ICD-10 R63.4
**Williams syndrome**	
ICD-10 Q93.82	
